# Different Types of Statins and All-Cause Mortality during Anticoagulation for Venous Thromboembolism: Validation Study from RIETE Registry

**DOI:** 10.1055/s-0040-1716734

**Published:** 2020-09-17

**Authors:** Carmine Siniscalchi, José M. Suriñach, Adriana Visonà, José L. Fernández-Reyes, Covadonga Gómez-Cuervo, Peter Verhamme, Pablo J. Marchena, Dominique Farge-Bancel, Jorge Moisés, Manuel Monreal, and the RIETE Investigators

**Affiliations:** 1 Department of Internal and Emergency Medicine, Angiology Unit, Parma University Hospital, Parma, Italy; 2 Department of Internal Medicine, Hospital Universitario Vall d'Hebron, Barcelona, Spain; 3 Department of Vascular Medicine, Ospedale Castelfranco Veneto, Castelfranco Veneto, Italy; 4 Department of Internal Medicine, Complejo Hospitalario de Jaén, Jaén, Spain; 5 Department of Internal Medicine, Hospital Universitario 12 de Octubre, Madrid, Spain; 6 Vascular Medicine and Haemostasis, University of Leuven, Leuven, Belgium; 7 Department of Internal Medicine and Emergency, Parc Sanitari Sant Joan de Déu-Hospital General, Barcelona, Spain; 8 Department of Internal Medicine and Pathology, Hôpital Saint-Louis, Paris, France; 9 Department of Pneumonology, Hospital Universitario Clínic de Barcelona, Barcelona, Spain; 10 Department of Internal Medicine, Hospital Germans Trias i Pujol, Badalona, Barcelona, Universidad Católica de Murcia, Spain

**Keywords:** atorvastatin, simvastatin, rosuvastatin, venous thromboembolism, death

## Abstract

**Introduction** We previously reported that during the course of anticoagulation for venous thromboembolism (VTE) patients using statins were at a lower risk to die than nonusers.

**Methods** We used the R egistro I nformatizado E nfermedad T rombo E mbólica (RIETE) registry to validate our previous findings in a subsequent cohort of patients and to compare the risk of death according to the use of different types of statins.

**Results** From January 2018 to December 2019, 19,557 patients with VTE were recruited in RIETE. Of them, 4,065 (21%) were using statins (simvastatin, 1,406; atorvastatin, 1,328; rosuvastatin, 246; and others, 1,085). During anticoagulation (192 vs.182 days, for statin and no statin users respectively), 500 patients developed a VTE recurrence, 519 suffered major bleeding, and 1,632 died (fatal pulmonary embolism [PE], 88 and fatal bleeding, 78). On multivariable analysis, statin users were at a lower risk to die (hazard ratio [HR] = 0.68; 95% confidence interval [CI]: 0.59–0.79) than nonusers. When separately analyzing the drugs, on multivariable analysis, patients using simvastatin (HR = 0.64; 95% CI: 0.52–0.80), atorvastatin (HR 0.72; 95% CI: 0.58–0.89), or other statins (HR = 0.67; 95% CI: 0.52–0.87) were at a lower risk to die than nonusers. For those using rosuvastatin, difference was not statistically significant (HR = 0.77; 95% CI: 0.50–1.19), maybe due to the sample size.

**Conclusion** Our data validate previous findings and confirm that VTE patients using statins at baseline are at a lower risk to die than nonusers. No statistically differences were found according to type of statins.

## Introduction

The impact of statins on survival in patients with coronary, cerebrovascular, or peripheral artery disease has been extensively studied, 1 2 3 4 5 6 but only few studies have evaluated their influence on mortality in patients receiving anticoagulation for venous thromboembolism (VTE). 7 8 9 Several prior studies suggest a lower rate of VTE recurrences in statin users during anticoagulation for VTE. The JUPITER study reported a 43% reduction with the use of rosuvastatin 7 but did not provide data on mortality. 7 A recent meta-analysis and metaregression of randomized controlled trials 8 9 demonstrated that statin therapy affects coagulation factors and thrombin generation, but its relationship whit the risk to die during anticoagulation in statin users has not been consistently studied. One study found that patients with acute pulmonary embolism (PE) who were using statins at baseline had half the risk to die than nonusers, 10 and a case-control study also found that statin users presenting with VTE were at a lower risk for death than nonusers. 11 However, both studies were based on population registries of records linked with hospital discharge records where the diagnosis of VTE is based on diagnostic codes, and mortality was a secondary outcome. In a recent R egistro I nformatizado E nfermedad T rombo E mbólica (RIETE) study that included 32,062 patients with a first episode of VTE, we observed that those using statins (22% of the whole cohort) demonstrated a 38% lower mortality compared with nonusers. 12

The RIETE registry is an ongoing, multicenter, observational registry of consecutive patients with objectively confirmed acute VTE (ClinicalTrials.gov identifier: NCT02832245). Data from this registry have been used to evaluate outcomes after acute VTE such as the frequency of recurrent VTE, major bleeding or mortality, and risk factors for these outcomes. 13 14 15 16 17 18 The rationale and methodology of RIETE have been published elsewhere. 19 In the current analysis, we aimed to validate our prior observations in a subsequent cohort of patients with VTE and to compare the mortality risk according to the type of statins.

## Methods

### Inclusion Criteria

Consecutive patients with acute, symptomatic deep vein thrombosis (DVT) or PE confirmed by objective tests (compression ultrasonography or contrast venography for DVT; helical computed tomography [CT] scan of the chest, ventilation-perfusion lung scintigraphy, or angiography for PE) were enrolled in RIETE. Patients were excluded if they were currently participating in a blinded/double-blind therapeutic clinical trial. All patients (or their relatives) provided written- or oral-informed consent for participation in the registry, in accordance with local ethics committee requirements.

### Study Design

For this study, only patients with available information on the use of statins at baseline and who were not previously included in the prior study were considered. Thus, we included all patients with acute symptomatic VTE recruited from February 2018 to December 2019. The major study outcome was all-cause mortality. Secondary outcomes were fatal PE and fatal bleeding. Bleeding events were classified as major if they were overt and required a transfusion of 2 units of blood or more, or were retroperitoneal, spinal, or intracranial. Fatal PE, in the absence of autopsy, was defined as any death appearing <10 days after PE diagnosis, in the absence of any alternative cause of death. Fatal bleeding was defined as any death occurring <10 days after a major bleeding episode, in the absence of any alternative cause of death.

### Study Variables

The following parameters are recorded in RIETE: patient's baseline characteristics; clinical status including any coexisting or underlying conditions such as chronic heart or lung disease, recent major bleeding, anemia, or renal insufficiency; risk factors for VTE; the treatment received upon VTE diagnosis; concomitant drugs; and the outcomes during the course of therapy. Immobilized patients were defined as nonsurgical patients who had been immobilized (i.e., total bed rest with or without bathroom privileges) for ≥4 days in the 2-month period prior to VTE diagnosis. Surgical patients were defined as those who had undergone an operation in the 2 months prior to VTE. Active cancer was defined as newly diagnosed cancer (<3 months before) or when receiving antineoplastic treatment of any type (i.e., surgery, chemotherapy, radiotherapy, hormonal, support therapy, or combined therapies). Recent bleeding was defined as major bleeding <30 days prior to VTE. Anemia was defined as hemoglobin levels <13 g/dL for men and <12 g/dL for women.

### Treatment and Follow-up

Patients were managed according to the clinical practice of each participating hospital (i.e., there was no standardization of treatment). The type, dose, and duration of anticoagulant therapy were recorded. After VTE diagnosis, all patients were followed-up in the outpatient clinic for at least 3 months, and thereafter as long as possible. During each visit, any signs or symptoms suggesting VTE recurrences or bleeding complications were noted. Each episode of clinically suspected recurrent VTE was investigated by repeat compression ultrasonography, lung scanning, helical CT scan, or pulmonary angiography as appropriate. The RIETE investigators assessed mortality using medical record review, and proxy interviews when necessary. Most outcomes were classified as reported by the clinical centers. However, if staff at the coordinating center were uncertain how to classify a reported outcome, the event was reviewed by a central adjudicating committee (< 10% of events).

### Statistical Analysis

Categorical variables were compared using the Chi-square test (two-sided) and Fisher's exact test (two-sided). Continuous variables were compared using Student's *t* -test. Incidence rates were calculated as events per 100 patient-years of follow-up and compared between patients receiving and those not receiving statins at baseline, using the hazard ratios (HRs) and corresponding 95% confidence intervals (CIs). We compared the cumulative mortality rates between statin users and nonusers, and also according to the use of different types of statins with time-to-event methods. Mortality was assessed with Cox's proportional hazard model. Time zero was the date of VTE and censoring occurred at the time of last follow-up. Covariates included in the adjusted model were those for which a statistically significant difference (a threshold *p* -value of 0.1 was set to assess significance of differences) was found, and a backward selection was used for the covariate selection in the multivariable model. Statistical analyses were conducted with SPSS for Windows Release (version 20, SPSS Inc. Chicago, Illinois, United States).

## Results

**Fig. 1:**
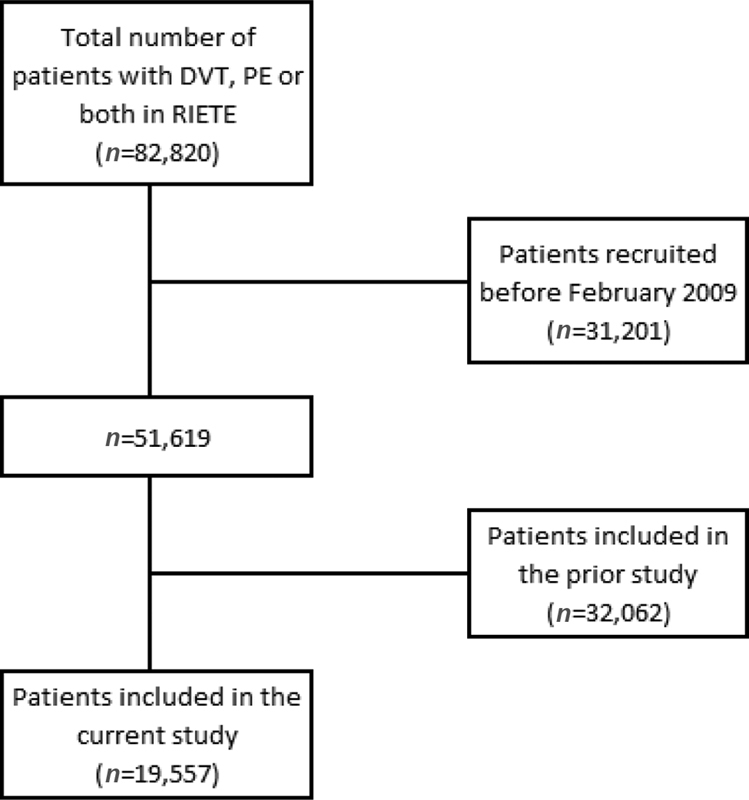
Patients with VTE who were recruited in R egistro I nformatizado E nfermedad T rombo E mbólica (RIETE). DVT, deep vein thrombosis; PE, pulmonary embolism.

From January 2018 to December 2019, 19,557 patients with VTE were recruited in RIETE ( Fig. 1 ). Of them, 4,065 (21%) were using statins at baseline (simvastatin, 1,406; atorvastatin, 1,328; rosuvastatin, 246; and other statins, 1,085). Compared with nonusers, statin users were 9 years older and were more likely to have comorbidities such as chronic heart failure, lung disease, hypertension, diabetes, prior myocardial infarction, prior ischemic stroke, peripheral artery disease, anemia, or renal insufficiency ( Table 1 ). Statin users were also more likely to be using antiplatelet agents or corticosteroids at baseline and had lower levels of total- and low-density lipoprotein (LDL)-cholesterol at baseline. Similar rates of active cancer were found in both subgroups.

**Table 1:** Clinical characteristics in 19,557 patients with VTE, according to use of statins at baseline

	Statins users	Non-users	Odds ratio (95% CI)
Patients ( *n* )	4,065	15,492	
Clinical characteristics			
Age (y) Mean ± SD	73 ± 11	64 ± 18 ^c^	<0.0001
Male	2,013 (50)	7,800 (50)	0.97 (0.90–1.04)
Body weight (kg) Mean ± SD	78 ± 15	77 ± 17 ^c^	<0.0001
Comorbidities			
Chronic lung disease	605 (15)	1,626 (10) ^c^	1.49 (1.35–1.65)
Chronic heart failure	441 (11)	818 (5.3) ^c^	2.18 (1.93–2.46)
Recent major bleeding	91 (2.2)	331 (2.1)	1.05 (0.83–1.33)
Hypertension	2,995 (74)	5,669 (37) ^c^	4.85 (4.49–5.24)
Diabetes	1,151 (28)	1,620 (10) ^c^	3.38 (3.11–3.68)
Current smoking	387 (9.5)	2,053 (13) ^c^	0.69 (0.61–0.77)
Prior myocardial infarction	700 (17)	479 (3.6) ^c^	5.60 (4.96–6.33)
Prior ischemic stroke	480 (12)	578 (4.4) ^c^	2.96 (2.61–3.36)
Peripheral artery disease	298 (7.5)	357 (2.7) ^c^	2.87 (2.45–3.37)
Concomitant therapies			
Antiplatelets	1,463 (36)	1,747 (11) ^c^	4.42 (4.08–4.80)
Corticosteroids	396 (9.7)	1,241 (8.0) ^c^	1.24 (1.10–1.40)
Risk factors for VTE			
Immobilization ≥4 days	777 (19)	2,800 (18)	1.07 (0.98–1.17)
Active cancer	607 (15)	2,536 (16) ^a^	0.90 (0.81–0.99)
Surgery	379 (9.3)	1,475 (9.5)	0.98 (0.87–1.10)
Estrogen use	82 (2.0)	863 (5.6) ^c^	0.35 (0.28–0.44)
Pregnancy or postpartum	4 (0.10)	176 (1.1) ^c^	0.09 (0.03–0.23)
None of the above	2,461 (61)	8,663 (56) ^c^	1.21 (1.13–1.30)
Initial VTE presentation			
Pulmonary embolism	2,403 (59)	8,511 (55%) ^c^	1.19 (1.11–1.27)
In patients with PE			
SBP levels <100 mm Hg	158 (6.6)	612 (7.2)	0.91 (0.76–1.09)
Heart rate >110 bpm	330 (14)	1,253 (15)	0.90 (0.79–1.03)
Sat O _2_ levels <90%	365 (30)	1,052 (27) ^a^	1.16 (1.00–1.33)
Blood tests			
Anemia	1,344 (33)	4,848 (31%) ^a^	1.08 (1.01–1.17)
Platelet count <100,000/μL	105 (2.6)	370 (2.4)	1.08 (0.87–1.35)
CrCl levels <50 mL/min	1,075 (26)	2,771 (18) ^c^	1.65 (1.52–1.79)
Cholesterol levels (mg/dL) Mean ± SD	170 ± 43	182 ± 67 ^c^	0.000
LDL-cholesterol levels (mg/dL) Mean ± SD	101 ± 85	120 ± 186 ^c^	0.001

Abbreviations: CI, confidence intervals; CrCl, creatinine clearance; LDL, low-density lipoprotein; PE, pulmonary embolism; SD, standard deviation; VTE, venous thromboembolism.

Notes: Age is presented as mean (±standard deviation). Other numbers presented as *n* (%).

Differences between statin users and nonusers: ^a^ *p* <0.05; ^b^ *p* <0.01; ^c^ *p* <0.001.

Most patients in both subgroups were initially treated with low molecular weight heparin (LMWH), at similar daily doses ( Table 2 ). Among the remaining patients, statin users were less likely to receive direct oral anticoagulants (DOACs) than nonusers. For long-term therapy, statin users were more likely to receive vitamin-K antagonists and less likely to receive DOACs. Among all, 63 patients did not receive anticoagulant therapy, and were excluded from the analysis. Median duration of anticoagulant therapy was slightly longer in statin users: 192 (interquartile range [IQR]: 104–376 days) versus 182 (IQR: 102–345) days, respectively.

**Table 2:** Treatment strategies

	Statins users	Non users	OR (95% CI)
Patients ( *n* )	4,065	15,492	
Initial therapy			
LMWH	3,522 (87)	12,865 (83) ^c^	1.32 (1.20–1.46)
Mean LMWH doses (IU/kg/day)	168 ± 45	169 ± 44	0.085
Unfractionated heparin	167 (4.1)	759 (4.9) ^a^	0.83 (0.70–0.99)
Fondaparinux	77 (1.9)	443 (2.9) ^c^	0.66 (0.51–0.84)
Direct oral anticoagulants	171 (4.2)	957 (6.2) ^c^	0.67 (0.56–0.79)
Thrombolytics	57 (1.4)	210 (1.4)	1.03 (0.77–1.39)
No anticoagulant therapy	17 (0.42)	46 (0.30)	1.41 (0.81–2.46)
Vena cava filter	120 (3.0)	424 (2.7)	1.08 (0.88–1.33)
Long-term therapy			
Vitamin-K antagonists	2,112 (52)	7,325 (47) ^c^	1.21 (1.13–1.29)
LMWH	1,167 (29)	4,553 (29)	0.97 (0.90–1.04)
Mean LMWH doses (IU/kg/day)	149 ± 44	152 ± 45 ^a^	0.030
Fondaparinux	19 (0.47)	129 (0.83) ^a^	0.56 (0.35–0.91)
Direct oral anticoagulants	664 (16)	2,998 (19) ^c^	0.81 (0.74–0.89)
Duration of anticoagulant therapy			
Mean days (±SD)	313 ± 352	292 ± 364 ^b^	0.001
Median days (IQR)	192 (104–376)	182 (102–345)	0.000
Over 6 months	2,193 (54)	7,848 (51) ^c^	1.14 (1.06–1.22)
Over 12 months	1,087 (27)	3,515 (23) ^c^	1.24 (1.15–1.35)

**Abbreviations:** CI, confidence intervals; IQR, interquartile range; IU, international units; LMWH, low molecular weight heparin; OR, odds ratio; SD, standard deviation.

Notes: Values are presented as mean (±standard deviation), median (IQR), and *n* (%).

Differences between statin users and nonusers: ^a^ *p* < 0.05; ^b^ *p* < 0.01; ^c^ *p* < 0.001.

During the course of anticoagulant therapy, 500 patients developed VTE recurrences, 519 had major bleeding and 1,632 died (fatal PE, 88 and fatal bleeding 78). In nonadjusted analyses, statin users had a significantly lower mortality rate (HR = 0.80; 95% CI: 0.71–0.91), a nonsignificantly lower rate of VTE recurrences (HR = 0.84; 95% CI: 0.67–1.04), and a higher rate of major bleeding (HR = 1.27; 95% CI: 1.04–1.54) than nonusers ( Table 3 ). Statin users had a nonsignificant lower rates of fatal PE (HR = 0.67; 95% CI: 0.37–1.17) and a similar rate of fatal bleeding (HR = 1.07; 95% CI: 0.62–1.79; Table 4 ).

**Table 3:** Clinical outcomes during the course of anticoagulant therapy

	Statins users		Nonusers		Hazard ratio (95% CI)
	*n*	Events per 100 patient-years	*n*	Events per 100 patient-years	
Patients ( *n* )	4,065		15,492		
Recurrent PE	46	1.34 (0.99–1.77)	185	1.51 (1.30–1.74)	0.89 (0.64–1.22)
Recurrent DVT	53	1.55 (1.18–2.02)	238	1.96 (1.72–2.22)	0.79 (0.59–1.06)
Recurrent VTE	95	2.81 (2.29–3.42)	405	3.36 (3.05–3.70)	0.84 (0.67–1.04)
Major bleeding	136	3.97 (3.34–4.68)	383	3.13 (2.83–3.46) ^a^	1.27 (1.04–1.54)
Site of major bleeding					
Gastrointestinal	47	1.36 (1.01–1.79)	139	1.13 (0.95–1.33)	1.20 (0.86–1.67)
Hematoma	24	0.69 (0.45–1.01)	86	0.70 (0.56–0.86)	0.99 (0.62–1.55)
Intracranial	29	0.84 (0.57–1.19)	68	0.55 (0.43–0.69)	1.52 (0.97–2.33)
Myocardial infarction	8	0.23 (0.11–0.44)	19	0.15 (0.10–0.24)	1.50 (0.62–3.37)
Ischemic stroke	22	0.64 (0.41–0.95)	55	0.45 (0.34–0.58)	1.43 (0.86–2.32)
Death	300	8.64 (7.70–9.66)	1,332	10.8 (10.2–11.4) ^c^	0.80 (0.71–0.91)
Causes of death					
Pulmonary embolism	14	0.40 (0.23–0.66)	74	0.60 (0.47–0.75)	0.67 (0.37–1.17)
Bleeding	18	0.52 (0.32–0.80)	60	0.48 (0.37–0.62)	1.07 (0.62–1.79)
Respiratory failure	16	0.46 (0.27–0.73)	96	0.78 (0.63–0.94) ^a^	0.59 (0.34–0.99)
Sudden, unexpected	7	0.20 (0.09–0.40)	21	0.17 (0.11–0.25)	1.19 (0.47–2.72)
Disseminated malignancy	125	3.60 (3.01–4.27)	548	4.43 (4.07–4.81) ^a^	0.81 (0.67–0.98)
Infection	22	0.63 (0.41–0.94)	98	0.79 (0.65–0.96)	0.80 (0.49–1.25)
Multiorganic failure	17	0.49 (0.29–0.77)	85	0.69 (0.55–0.85)	0.71 (0.41–1.18)
Heart failure	15	0.43 (0.25–0.70)	51	0.41 (0.31–0.54)	1.05 (0.57–1.83)
Bronchoaspiration	6	0.17 (0.07–0.36)	38	0.31 (0.22–0.42)	0.56 (0.22–1.27)
Ischemic stroke	4	0.12 (0.04–0.28)	8	0.06 (0.03–0.12)	1.78 (0.47–5.89)
Myocardial infarction	2	0.06 (0.01–0.19)	7	0.06 (0.02–0.11)	1.02 (0.14–4.57)

Abbreviations: CI, confidence intervals; DVT, deep vein thrombosis; PE, pulmonary embolism.

Note: Differences between statin users and nonusers: ^a^ *p* <0.05; ^b^ *p* <0.01; ^c^ *p* <0.001.

**Table 4:** Clinical outcomes during anticoagulation, according to the type of statin

	Simvastatin		Atorvastatin		Rosuvastatin		Others	
	*n*	Events per 100 patient-years	*n*	Events per 100 patient-years	*n*	Events per 100 patient-years	*n*	Events per 100 patient-years
Patients ( *n* )	1,406		1,328		246		1,085	
Recurrent DVT	20	1.67 (1.05–2.54)	14	1.38 (0.78–2.25)	2	1.09 (0.18–3.61)	17	1.68 (1.01–2.63)
Recurrent PE	17	1.41 (0.85–2.21)	16	1.58 (0.94–2.52)	2	1.09 (0.18–3.61)	11	1.07 (0.56–1.85)
Recurrent VTE	36	3.03 (2.16–4.15)	28	2.79 (1.89–3.98)	4	2.19 (0.70–5.29)	27	2.68 (1.80–3.85)
Major bleeding	36	3.00 (2.13–4.11)	53	5.19 (3.93–6.74) ^b^	14	7.93 (4.52–13.0) ^b^	33	3.20 (2.24–4.44)
Death	92	7.56 (6.13–9.23) ^c^	114	11.1 (9.16–13.2)	23	12.5 (8.12–18.5)	71	6.82 (5.37–8.56) ^c^
Causes of death								
Pulmonary embolism	3	0.25 (0.06–0.67)	7	0.68 (0.30–1.34)	1	0.54 (0.03–2.68)	3	0.29 (0.07–0.78)
Initial PE	1	0.08 (0.00–0.41) ^a^	6	0.58 (0.24–1.21)	1	0.54 (0.03–2.68)	3	0.29 (0.07–0.78)
Recurrent PE	2	0.16 (0.03–0.54)	1	0.10 (0.00–0.48)	0	–	0	–
Bleeding	2	0.16 (0.03–0.54) ^a^	8	0.78 (0.36–1.47)	1	0.54 (0.03–2.68)	7	0.67 (0.29–1.33)
Respiratory failure	7	0.58 (0.25–1.14)	5	0.48 (0.18–1.07)	1	0.54 (0.03–2.68)	3	0.29 (0.07–0.78)
Sudden, unexpected	3	0.25 (0.06–0.67)	4	0.39 (0.12–0.94)	0	–	0	–
Malignancy	47	3.86 (2.87–5.09)	38	3.69 (2.65–5.01)	11	5.98 (3.15–10.4)	29	2.79 (1.90–3.95) ^a^
Infection	11	0.90 (0.48–1.57)	9	0.87 (0.43–1.60)	0	–	2	0.19 (0.03–0.64) ^a^
Multiorganic failure	1	0.08 (0.00–0.41) ^b^	8	0.78 (0.36–1.47)	3	1.63 (0.42–4.44)	5	0.48 (0.18–1.07)
Heart failure	1	0.08 (0.00–0.41)	9	0.87 (0.43–1.60)	1	0.54 (0.03–2.68)	4	0.38 (0.12–0.93)
Bronchoaspiration	0	–	3	0.29 (0.07–0.79)	0	–	3	0.29 (0.07–0.78)
Ischemic stroke	0	–	3	0.29 (0.07–0.79)	0	–	1	0.10 (0.00–0.47)
Myocardial infarction	0	–	1	0.10 (0.00–0.48)	0	–	1	0.10 (0.00–0.47)

Abbreviations: DVT, deep vein thrombosis; PE, pulmonary embolism; VTE, venous thromboembolism.

Notes: Results expressed as number of events per 100 patient-years.

Differences between subgroups: ^a^ *p* <0.05; ^b^ *p* <0.01; ^c^ *p* <0.001.

Compared with nonstatin users, patients using simvastatin (HR = 0.72; 95% CI: 0.58–0.88) or other statins (HR = 0.70; 95% CI: 0.55–0.89) had a lower mortality rate, but there were no significant differences in those using atorvastatin (HR = 1.00; 95% CI: 0.83–1.21) or rosuvastatin (HR = 1.10; 95% CI: 0.73–1.66; Table 5 ).

**Table 5:** Multivariable analyses for all-cause death

	Univariable	Multivariable
	1,632	
Statins users (yes)	0.82 (0.73–0.93) a	0.68 (0.59–0.79) b
Nonusers	Ref. a	Ref. ^b^
Simvastatin	0.72 (0.58–0.88) a	0.64 (0.52–0.80) b
Atorvastatin	1.00 (0.83–1.21)	0.72 (0.58–0.89) a
Rosuvastatin	1.10 (0.73–1.66)	0.77 (0.50–1.19)
Others	0.70 (0.55–0.89) a	0.67 (0.52–0.87) a

Notes: Results are expressed as hazard ratio (95% confidence intervals).

Variables entering in the multivariate analysis: patients' age, body weight, chronic lung disease, chronic heart failure, recent major bleeding, recent surgery, recent immobility ≥4 days, estrogen use, pregnancy, active cancer, prior VTE, initial VTE presentation, anemia, platelet count <100,000/μL, creatinine clearance levels, diabetes, current smoking, prior myocardial infarction, prior ischemic stroke, peripheral artery disease, concomitant therapy with antiplatelet drugs, and concomitant therapy with corticosteroids.

a *p* < 0.01.

b *p* < 0.001.

**Fig. 2:**
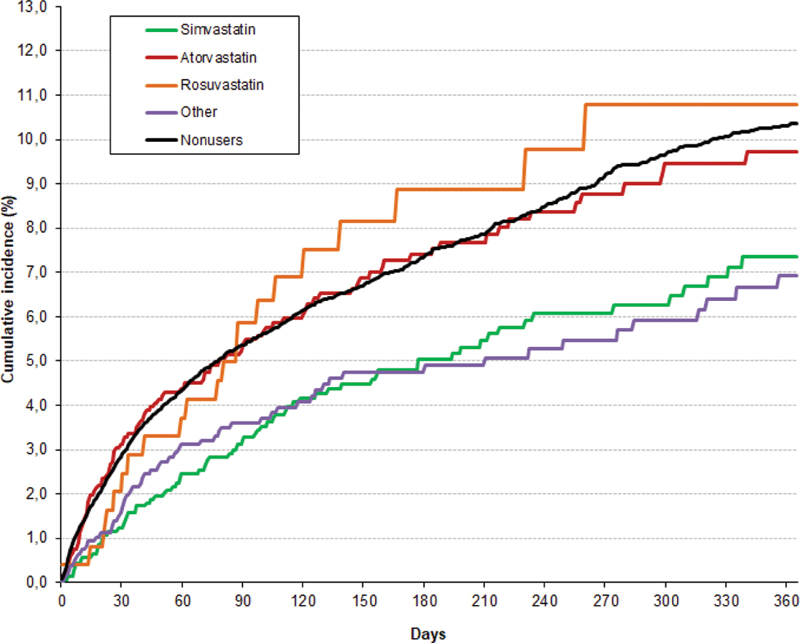
Cumulative mortality rates according to type of statin used.

On multivariable analysis (after adjusting for patients' age, body weight, chronic lung or heart disease, diabetes, prior artery disease, recent major bleeding, risk factors for VTE, initial VTE presentation, anemia, platelet count, renal function, and concomitant therapy with antiplatelet agents or corticosteroids), statin users had a significantly lower risk to die (HR = 0.68; 95% CI: 0.59–0.79) compared with nonusers. Concerning the different statins, patients using simvastatin (HR = 0.64; 95% CI: 0.52–0.80), atorvastatin (HR = 0.72; 95% CI: 0.58–0.89), or other statins (HR = 0.67; 95% CI: 0.52–0.87) were at a lower risk to die than nonusers. Patients using rosuvastatin had a nonsignificantly lower risk (HR = 0.77; 95% CI: 0.50–1.19), as shown in Table 5 and Fig. 2 .

## Discussion

Our data confirm that VTE patients using statins at baseline are at a lower risk to die than nonusers, as previously reported. One out of four patients (26%) in our cohort were using statins at baseline, and during anticoagulation, they had a 20% lower rate of mortality rate despite being older and more likely to have comorbidities. Multivariable adjusted analyses confirmed that this decrease was independent from several potential confounders, and didn't show differences between types of statins.

Statins are widely used in patients with cardiovascular risk factors 20 and their influence on survival in these patients has been largely reported. 1 2 3 4 5 Few studies investigated their influence on mortality in patients receiving anticoagulation for VTE. In our previous study, we found that statin users ( *n* = 7,085) had a 38% reduction in all-cause mortality compared with nonusers (adjusted HR = 0.62; 95% CI: 0.48–0.79). 12 The current study validates and confirms these results with a similar decrease (adjusted HR = 0.68; 95% CI: 0.59–0.79). Our findings are based on a cohort of patients with symptomatic, objectively proven diagnosis of VTE, not on an administrative database where the diagnosis of VTE was based on less specific diagnostic codes and hospital discharge records as in some other studies on this topic. 10 11

Interestingly however, we failed to find differences between types of statins. In particular, we failed to find differences for those statins that lower LDL-C or total cholesterol levels more potently. Although we cannot provide a biologically plausible explanation, these findings might be due to two reasons. First, the observed lower mortality rate is not due to the effect of statins on LDL-C or total cholesterol levels, but rather to other “pleiotropic” mechanisms, such as platelet inhibition, reduction of inflammation, reduction of C-reactive protein, increased production of nitric oxide, or downregulation of the coagulation cascade. 6 21 This is supported by meta-analysis and metaregression of randomized controlled trials 8 and randomized clinical trial (START) 9 and several clinical studies demonstrating that statin therapy either with rosuvastatin, 21 simvastatin, 22 atorvastatin, 23 24 or cerivastatin 25 affects coagulation factors and thrombin generation. In our study, no differences in levels of total or LDL cholesterol at baseline were observed and this can be in line with this hypothesis. Second, also in patients with cardiovascular risk factors, statin use may decrease mortality not only through their lipid-lowering effect but also through anti-inflammatory effects. 26 Results from the JUPITER trial demonstrated that the risk of cardiovascular events decreased by 79% in rosuvastatin users who achieved the targets of high-sensitivity C-reactive protein less than 1 mg/L and LDL cholesterol less than 1.8 mmol/L (HR = 0.21, 95% CI: 0.09–0.51). This effect was less prominent in patients who achieved only the target of LDL cholesterol (HR = 0.49, 95% CI: 0.37–0.66). These results suggest that the anti-inflammatory effects are a part of the complex mechanism by which statins exercise their activities on cardiovascular events and probably on VTE events. 27 28

The RIETE registry provides data on the treatment of VTE in the real world with unselected population. Data from our patients reflect routine, unmonitored medical practice involving a broad spectrum of patients with VTE. It can, therefore, provide insights into the natural history of VTE in the special subgroup populations and to be hypothesis generating. The same reasons may result in some limitations. First, RIETE is an observational study with no randomization to statins versus no statins users, although the number of patients included was high. Thus, some nonmeasured variables, such as the reason why patients were using statins, could lead to a possible bias. Second, residual confounding may remain in the findings, as certain potential confounding variables may not be recorded on the database or may not be recorded in sufficient detail to completely remove their confounding effect. Third, in RIETE, we do not gather information on the use of statins over time, but only at baseline. However, since anticoagulant therapy is not a contraindication to stop the use of statins, we may assume (with limitations) that most patients did continue with this therapy. Fourth, uncontrolled healthy-user or healthy-adherer bias cannot be excluded either. But statin users in our cohort were 10 years older and had more comorbidities than nonusers. Although we provided adjusted results for several of the outcomes, we cannot exclude the possibility of residual differences in the two groups, and hence residual confounding. Fifth, we cannot be excluded a “social effect” where statin users may have a better health care. Finally, almost half of the mortality is cancer related ( Table 3 ), and this drives the reduced mortality in users. In contrast, cardiovascular mortality (stroke, myocardial infraction, and heart failure) is rather low. Hence, if there is a causal impact of statins on mortality in VTE patients, this is unlikely to be explained by major adverse cardiovascular events.

## Conclusion

In conclusion, our findings confirm that in patients receiving anticoagulant therapy for VTE the use of statins was associated with a 20% decrease in mortality, irrespective of different statin type. Intervention studies specifically designed to confirm our findings in patients receiving anticoagulant therapy for VTE are warranted.
