# Supplementary material for: Different Types of Statins and All-Cause Mortality during Anticoagulation for Venous Thromboembolism: Validation Study from RIETE Registry
Source: TH Open. 2020 Sep 17;4(3):e236–44. doi: 10.1055/s-0040-1716734 (PMC7498305; doi:10.1055/s-0040-1716734)
Supplement: Supplementary file 1 — Supplementary Material [file 10-1055-s-0040-1716734-s200015.pdf]

Supplementary Material

Members of the RIETE Group are as follows:

Spain: Adarraga M.D., Agud M., Aibar J., Aibar M.A., Alfonso J., Amado C., Aramberri M., Arcelus J.I., Baeza C., Ballaz A., Barba R., Barbagelata C., Barrón M., Barrón-Andrés B., Blanco-Molina A., Botella E., Camon A.M., Campos S., Cañas I., Casado I., Castro J., Criado J., de Ancos C., de Miguel J., del Toro J., Demelo-Rodríguez P., Díaz-Pedroche C., Díaz-Peromingo J.A., Díez-Sierra J., Domínguez I.M., Encabo M., Escribano J.C., Falgá C., Farfán A.I., Fernández de Roitegui K., Fernández-Capitán C., Fernández-Reyes J.L., Fidalgo M.A., Flores K., Font C., Font L., Francisco I., Furest I., Gabara C., Galeano-Valle F., García M.A., García-Bragado F., Gavín-Sebastián O., Gil-Díaz A., Gómez-Cuervo C., Gómez-Mosquera A.M., González-Martínez J., Grau E., Giménez-Suau M., Guirado L., Gutiérrez J., Hernández-Blasco L., Hernando E., Jara-Palomares L., Jaras M.J., Jiménez D., Joya M.D., Jou I., Lecumberri R., Lima J., Lobo J.L., López-Jiménez L., López-Miguel P., López-Núñez J.J., López-Reyes R., López-Sáez J.B., Lorente M.A., Lorenzo A., Loring M., Madridano O., Maestre A., Marchena P.J., Martín del Pozo M., Martín-Martos F., Martínez-García M.A., Mella C., Mellado M., Mercado M.I., Moisés J., Monreal M., Morales M.V., Muñoz-Blanco A., Muñoz M., Muñoz-Guglielmetti D., Muñoz-Rivas N., Nieto J.A., Núñez-Ares A., Núñez-Fernández M.J., Obispo B., Olivares M.C., Orcastegui J.L., Ortega-Recio M.D., Osorio J., Otalora S., Otero R., Parra P., Parra V., Pedrajas J.M., Pellejero G., Perez-Jacoiste A., Pesántez D., Porras J.A., Portillo J., Reig L., Riera-Mestre A., Rivas A., Rivera F., Rodríguez-Cobo A., Rodríguez-Matute C., Rogado J., Rosa V., Rubio C.M., Ruiz-Artacho P., Ruiz-Giménez N., Ruiz-Ruiz J., Ruiz-Sada P., Sahuquillo J.C., Salgueiro G., Sampériz A., Sánchez-Muñoz-Torrero J.F., Sancho T., Sigüenza P., Soler S., Suárez S., Suriñach J.M., Torres M.I., Tolosa C.; Trujillo-Santos J., Uresandi F., Usandizaga E., Valle R., Vela J.R., Vidal G., Villares P., Zamora C.

Argentina: Gutiérrez P., Vázquez F.J.

Belgium: Vanassche T., Vandenbriele C., Verhamme P.

Czech Republic: Hirmerova J., Malý R.

Ecuador: Falconí A., Salgado E.

France: Benzidia I., Bertoletti L., Bura-Riviere A., Crichi B., Debourdeau P., Espitia O., Farge-Bancel D., Helfer H., Mahé I., Moustafa F., Poenou G.

Germany: Schellong S.

Israel: Braester A., Brenner B., Tzoran I.

Italy: Bilora F., Brandolin B., Ciammaichella M., Colaizzo D., Di Micco P., Grandone E., Mastroiacovo D., Maida R., Mumoli N., Pace F., Pesavento R., Pomero F., Prandoni P., Quintavalla R., Rocci A., Siniscalchi C., Tufano A., Visonà A., Zalunardo B.

Latvia: Kalejs R.V., Make K., Skride A.

Portugal: Ferreira M., Fonseca S., Martins F., Meireles J.

Republic Of Macedonia: Bosevski M.

Switzerland: Bounameaux H., Mazzolai L.

United States: Caprini J.A., Tafur A.J., Weinberg I., Wilkins H.

Vietnam: Bui H.M.
